# Supplementary material for: MetaRibo-Seq measures translation in microbiomes
Source: Nat Commun. 2020 Jun 29;11:3268. doi: 10.1038/s41467-020-17081-z (PMC7324362; doi:10.1038/s41467-020-17081-z)
Supplement: Supplementary file 10 — Supplementary Data 7 [file 41467_2020_17081_MOESM10_ESM.zip › File2/Confidence_VeryHigh_Taxonomy/29768_out.krona.html]

Javascript must be enabled to view this page.

members
magnitude
magnitudeUnassigned
count
unassigned
taxon
rank

29768\_out

337

superkingdom
337
2

337
976
phylum

class
337
200643

order
171549
337

family
815
337

816
41

SRS011239\_contig\_number\_contig-100\_8071.125182SRS013638\_contig\_number\_contig-100\_3062.126601SRS013800\_contig\_number\_contig-100\_2678.158997SRS013940\_contig\_number\_contig-100\_5258.127420SRS015217\_contig\_number\_30879SRS015782\_contig\_number\_contig-100\_4289.253930SRS015890\_contig\_number\_contig-100\_7424.145602SRS016056\_contig\_number\_2077SRS016541\_contig\_number\_contig-100\_7915.7915SRS016897\_contig\_number\_contig-100\_14056.14057SRS016954\_contig\_number\_contig-100\_3120.145757SRS017103\_contig\_number\_contig-100\_4458.195444SRS018049\_contig\_number\_contig-100\_3596.21945SRS019178\_contig\_number\_contig-100\_6472.6473SRS019286\_contig\_number\_contig-100\_2278.51411SRS020508\_contig\_number\_1045SRS024331\_contig\_number\_contig-100\_3884.184076SRS024625\_contig\_number\_contig-100\_5883.102811SRS046068\_contig\_number\_contig-100\_2020.43702SRS050998\_contig\_number\_contig-100\_10909.90688SRS052027\_contig\_number\_contig-100\_6937.104011SRS052078\_contig\_number\_contig-100\_4173.31833SRS058770\_contig\_number\_23758SRS063489\_contig\_number\_contig-100\_4227.56081SRS064757\_contig\_number\_contig-100\_20542.72988SRS065176\_contig\_number\_contig-100\_2453.103059SRS075341\_contig\_number\_contig-100\_4452.138525SRS075821\_contig\_number\_contig-100\_4159.129454SRS077991\_contig\_number\_contig-100\_8122.8123SRS078176\_contig\_number\_contig-100\_21065.60603SRS101268\_contig\_number\_contig-100\_4388.18307SRS1041032\_contig\_number\_contig-100\_21722.58739SRS1041139\_contig\_number\_contig-100\_3864.36685SRS104636\_contig\_number\_contig-100\_2384.175745SRS1055069\_contig\_number\_contig-100\_3704.88545SRS1055076\_contig\_number\_contig-100\_2961.92250SRS143181\_contig\_number\_contig-100\_19628.64357SRS147039\_contig\_number\_contig-100\_6711.6712SRS147919\_contig\_number\_contig-100\_7338.93329SRS147977\_contig\_number\_contig-100\_4103.75833SRS892882\_contig\_number\_contig-100\_3796.86950
337
genus


SRS019397\_contig\_number\_1075SRS022137\_contig\_number\_17717SRS045244\_contig\_number\_2040
3
2292957
species

species
19

SRS014736\_contig\_number\_7358SRS018575\_contig\_number\_4782SRS045826\_contig\_number\_16270SRS046810\_contig\_number\_2SRS048870\_contig\_number\_4306SRS052576\_contig\_number\_contig-100\_36.15545SRS054905\_contig\_number\_15501SRS054956\_contig\_number\_3921SRS056519\_contig\_number\_791SRS098717\_contig\_number\_12189SRS1041138\_contig\_number\_9296SRS1041141\_contig\_number\_9291SRS104485\_contig\_number\_289SRS142923\_contig\_number\_20768SRS143523\_contig\_number\_10173SRS143991\_contig\_number\_24875SRS893259\_contig\_number\_5254SRS893295\_contig\_number\_10893SRS893373\_contig\_number\_23701
1739517

1

SRS150029\_contig\_number\_12505
310298
species

3

SRS077335\_contig\_number\_2771SRS098881\_contig\_number\_22286SRS143148\_contig\_number\_6748
820
species

6

SRS015694\_contig\_number\_15525SRS020869\_contig\_number\_29508SRS055982\_contig\_number\_15175SRS063190\_contig\_number\_10180SRS098644\_contig\_number\_33292SRS893383\_contig\_number\_33424
469590
species

species
457390

SRS013476\_contig\_number\_40809SRS016132\_contig\_number\_5531SRS017622\_contig\_number\_120SRS017859\_contig\_number\_1359SRS018936\_contig\_number\_10141SRS018984\_contig\_number\_16553SRS020394\_contig\_number\_16581SRS020622\_contig\_number\_7085SRS023346\_contig\_number\_3780SRS043001\_contig\_number\_contig-100\_5240.58015SRS057480\_contig\_number\_8448SRS058268\_contig\_number\_349SRS064276\_contig\_number\_17759SRS076804\_contig\_number\_contig-100\_1347.126520SRS078419\_contig\_number\_20544SRS097889\_contig\_number\_16953SRS1041136\_contig\_number\_12909SRS140645\_contig\_number\_584SRS142980\_contig\_number\_6783SRS143780\_contig\_number\_1264SRS146764\_contig\_number\_26943SRS148721\_contig\_number\_39787SRS893279\_contig\_number\_11966SRS893327\_contig\_number\_11188
24

1

SRS077231\_contig\_number\_18889
2292944
species

3

SRS016335\_contig\_number\_48631SRS1041147\_contig\_number\_4935SRS893170\_contig\_number\_11765
1262750
species

72

SRS012849\_contig\_number\_13661SRS012969\_contig\_number\_contig-100\_4289.227508SRS013521\_contig\_number\_11954SRS013687\_contig\_number\_34590SRS013951\_contig\_number\_35169SRS014855\_contig\_number\_16920SRS015369\_contig\_number\_contig-100\_2949.55225SRS015579\_contig\_number\_24420SRS015663\_contig\_number\_26607SRS016095\_contig\_number\_contig-100\_1142.160973SRS016267\_contig\_number\_contig-100\_2684.86050SRS016753\_contig\_number\_contig-100\_2598.57888SRS017307\_contig\_number\_32941SRS018133\_contig\_number\_12447SRS018351\_contig\_number\_contig-100\_3295.101742SRS019601\_contig\_number\_contig-100\_3268.154821SRS019787\_contig\_number\_12443SRS019910\_contig\_number\_11994SRS020271\_contig\_number\_2760SRS022524\_contig\_number\_1246SRS022713\_contig\_number\_8393SRS023526\_contig\_number\_contig-100\_5882.200345SRS023583\_contig\_number\_1269SRS023914\_contig\_number\_15725SRS024075\_contig\_number\_15854SRS024132\_contig\_number\_contig-100\_468.253408SRS024388\_contig\_number\_11390SRS024435\_contig\_number\_contig-100\_2508.183855SRS044535\_contig\_number\_23632SRS045645\_contig\_number\_contig-100\_4593.157326SRS045739\_contig\_number\_4939SRS046369\_contig\_number\_16439SRS047433\_contig\_number\_contig-100\_3465.119237SRS049896\_contig\_number\_contig-100\_7273.166925SRS051031\_contig\_number\_14585SRS056273\_contig\_number\_33106SRS062654\_contig\_number\_21794SRS063040\_contig\_number\_17439SRS063518\_contig\_number\_15412SRS063813\_contig\_number\_5971SRS064557\_contig\_number\_10285SRS065397\_contig\_number\_7278SRS075078\_contig\_number\_8737SRS076756\_contig\_number\_21545SRS077086\_contig\_number\_4605SRS077127\_contig\_number\_5002SRS077194\_contig\_number\_31105SRS098571\_contig\_number\_62596SRS103987\_contig\_number\_contig-100\_3978.181471SRS1041039\_contig\_number\_contig-100\_7067.109914SRS1041095\_contig\_number\_6080SRS1041140\_contig\_number\_1189SRS1041164\_contig\_number\_5567SRS104975\_contig\_number\_contig-100\_3926.100286SRS105153\_contig\_number\_31383SRS1055049\_contig\_number\_14378SRS1055067\_contig\_number\_contig-100\_5282.91837SRS1055083\_contig\_number\_12716SRS1055099\_contig\_number\_contig-100\_4917.80675SRS140513\_contig\_number\_11050SRS143085\_contig\_number\_6153SRS143598\_contig\_number\_contig-100\_8916.126899SRS144506\_contig\_number\_35087SRS144603\_contig\_number\_13481SRS145308\_contig\_number\_907SRS147139\_contig\_number\_26517SRS147346\_contig\_number\_51257SRS148159\_contig\_number\_36538SRS148424\_contig\_number\_22162SRS148784\_contig\_number\_25558SRS148970\_contig\_number\_3982SRS893288\_contig\_number\_3798
2302926
species

1

SRS011302\_contig\_number\_19504
310297
species

species
47678

SRS049995\_contig\_number\_9145SRS075773\_contig\_number\_17888SRS893300\_contig\_number\_4131
3

species
2292282
5

SRS015694\_contig\_number\_contig-100\_5981.104430SRS018817\_contig\_number\_contig-100\_5325.80835SRS052027\_contig\_number\_contig-100\_3306.70096SRS062654\_contig\_number\_contig-100\_5230.108697SRS147346\_contig\_number\_contig-100\_6832.206168

species
821
2

SRS078665\_contig\_number\_9969SRS893342\_contig\_number\_1783


SRS077392\_contig\_number\_contig-100\_2105.2106
1
2292956
species

817

SRS014613\_contig\_number\_2138SRS015190\_contig\_number\_14968
2
species

371601
10

SRS017745\_contig\_number\_3138SRS019161\_contig\_number\_9234SRS074670\_contig\_number\_1008SRS075021\_contig\_number\_6459SRS077194\_contig\_number\_25998SRS077849\_contig\_number\_5559SRS097920\_contig\_number\_26205SRS1041031\_contig\_number\_contig-100\_513.112478SRS144362\_contig\_number\_22213SRS146813\_contig\_number\_25975
species

3

SRS013098\_contig\_number\_49326SRS051882\_contig\_number\_4741SRS893292\_contig\_number\_8932
2292951
species

species

SRS015794\_contig\_number\_5184SRS074964\_contig\_number\_368SRS104084\_contig\_number\_11398SRS1041134\_contig\_number\_13793
4
556259

species
246787
3

SRS020622\_contig\_number\_contig-100\_2010.43378SRS058723\_contig\_number\_contig-100\_1859.52678SRS1041092\_contig\_number\_15598

species
6

SRS021219\_contig\_number\_12701SRS045713\_contig\_number\_3861SRS050299\_contig\_number\_2792SRS057478\_contig\_number\_5061SRS101376\_contig\_number\_21028SRS1055095\_contig\_number\_162
818

species
1

SRS016018\_contig\_number\_4449
28111

species

SRS048164\_contig\_number\_4607
1
1796613

species

SRS011084\_contig\_number\_30615SRS012273\_contig\_number\_contig-100\_1511.245424SRS012902\_contig\_number\_1452SRS013215\_contig\_number\_259SRS013382\_contig\_number\_761SRS014287\_contig\_number\_13128SRS014412\_contig\_number\_15327SRS014459\_contig\_number\_37203SRS014683\_contig\_number\_13796SRS014979\_contig\_number\_36277SRS015065\_contig\_number\_26922SRS015264\_contig\_number\_4488SRS015431\_contig\_number\_10266SRS015578\_contig\_number\_9112SRS015854\_contig\_number\_23356SRS015960\_contig\_number\_16922SRS016203\_contig\_number\_9605SRS016381\_contig\_number\_2058SRS016840\_contig\_number\_2282SRS016989\_contig\_number\_contig-100\_3575.91174SRS017191\_contig\_number\_11264SRS017247\_contig\_number\_9425SRS017433\_contig\_number\_11248SRS017701\_contig\_number\_11530SRS017821\_contig\_number\_3496SRS018313\_contig\_number\_10528SRS018427\_contig\_number\_24055SRS018623\_contig\_number\_6803SRS018817\_contig\_number\_10263SRS019030\_contig\_number\_12108SRS019068\_contig\_number\_75356SRS019381\_contig\_number\_10450SRS019445\_contig\_number\_35184SRS019693\_contig\_number\_8532SRS019968\_contig\_number\_16107SRS021484\_contig\_number\_29189SRS022071\_contig\_number\_20343SRS023715\_contig\_number\_19482SRS023829\_contig\_number\_951SRS024549\_contig\_number\_1200SRS024663\_contig\_number\_3969SRS043411\_contig\_number\_3894SRS043701\_contig\_number\_9542SRS045004\_contig\_number\_15228SRS046712\_contig\_number\_2601SRS046717\_contig\_number\_8135SRS048981\_contig\_number\_6682SRS049773\_contig\_number\_35115SRS049959\_contig\_number\_6632SRS050026\_contig\_number\_11904SRS050422\_contig\_number\_contig-100\_1450.176549SRS050801\_contig\_number\_3637SRS050941\_contig\_number\_21977SRS052697\_contig\_number\_25897SRS053398\_contig\_number\_13726SRS053573\_contig\_number\_1005SRS053649\_contig\_number\_11941SRS054352\_contig\_number\_21404SRS056259\_contig\_number\_30783SRS057717\_contig\_number\_517SRS058070\_contig\_number\_945SRS062427\_contig\_number\_2808SRS064232\_contig\_number\_2878SRS065504\_contig\_number\_36448SRS075398\_contig\_number\_2850SRS075984\_contig\_number\_25756SRS077024\_contig\_number\_5532SRS077502\_contig\_number\_13002SRS077552\_contig\_number\_25352SRS078242\_contig\_number\_29545SRS097958\_contig\_number\_14464SRS098061\_contig\_number\_9261SRS098644\_contig\_number\_18826SRS098655\_contig\_number\_11486SRS098881\_contig\_number\_17037SRS1041037\_contig\_number\_16210SRS1041038\_contig\_number\_8626SRS1041112\_contig\_number\_5447SRS1041137\_contig\_number\_14502SRS104311\_contig\_number\_30345SRS104327\_contig\_number\_contig-100\_2748.45281SRS104400\_contig\_number\_43696SRS1055022\_contig\_number\_23028SRS1055034\_contig\_number\_3594SRS1055056\_contig\_number\_10024SRS142503\_contig\_number\_54610SRS142542\_contig\_number\_25300SRS143372\_contig\_number\_8605SRS143417\_contig\_number\_13461SRS143466\_contig\_number\_6144SRS143722\_contig\_number\_8927SRS143876\_contig\_number\_7057SRS144183\_contig\_number\_28304SRS144297\_contig\_number\_contig-100\_6259.83410SRS144537\_contig\_number\_25082SRS145497\_contig\_number\_3461SRS146812\_contig\_number\_50600SRS147022\_contig\_number\_340SRS147088\_contig\_number\_3847SRS147272\_contig\_number\_2194SRS147425\_contig\_number\_contig-100\_4073.65465SRS147653\_contig\_number\_2641SRS148196\_contig\_number\_31415SRS148253\_contig\_number\_1478SRS148319\_contig\_number\_12339SRS148511\_contig\_number\_17163SRS148817\_contig\_number\_12272SRS148874\_contig\_number\_16997SRS149075\_contig\_number\_13576SRS149181\_contig\_number\_1057SRS149244\_contig\_number\_11068SRS149784\_contig\_number\_27671SRS893231\_contig\_number\_2536SRS893253\_contig\_number\_1331SRS893256\_contig\_number\_1189SRS893285\_contig\_number\_contig-100\_4716.20300SRS893341\_contig\_number\_3596SRS893358\_contig\_number\_322SRS893369\_contig\_number\_9519SRS893378\_contig\_number\_12734SRS893382\_contig\_number\_903SRS971276\_contig\_number\_contig-100\_4686.175849
122
28116
